# The Effects of Unbleached and Bleached Nanocellulose on the Thermal and Flammability of Polypropylene-Reinforced Kenaf Core Hybrid Polymer Bionanocomposites

**DOI:** 10.3390/polym13010116

**Published:** 2020-12-30

**Authors:** Fatimah Athiyah Sabaruddin, M.T. Paridah, S. M. Sapuan, R. A. Ilyas, Seng Hua Lee, Khalina Abdan, Norkhairunnisa Mazlan, Adlin Sabrina Muhammad Roseley, H.P.S. Abdul Khalil

**Affiliations:** 1 Institute Tropical Forestry and Forest Product (INTROP), Universiti Putra Malaysia, Jalan Asam Jawa, Serdang Selangor 43400, Malaysia; atiyah88@gmail.com (F.A.S.); sapuan@upm.edu.my (S.M.S.); lee_seng@upm.edu.my (S.H.L.); 2 School of Industrial Technology, Universiti Sains Malaysia, Penang 11800, Malaysia; akhalilhps@gmail.com; 3 Advanced Engineering Materials and Composites Research Centre (AEMC), Department of Mechanical d Manufacturing Engineering, Faculty of Engineering, Universiti Putra Malaysia, Serdang Selangor 43400, Malaysia; 4 School of Chemical and Energy, Faculty of Engineering, Universiti Teknologi Malaysia, UTM Johor Bahru, Johor 81310, Malaysia; ahmadilyas@utm.my; 5 Centre for Advanced Composite Materials, Universiti Teknologi Malaysia, UTM Johor Bahru, Johor 81310, Malaysia; 6 Department of Aerospace Engineering, Faculty of Engineering, Universiti Putra Malaysia, Serdang Selangor 43400, Malaysia; norkhairunnisa@upm.edu.my; 7 Faculty of Forestry and Environment, Universiti Putra Malaysia, Serdang Selangor 43400, Malaysia; adlin@upm.edu.my

**Keywords:** lignin, sulfation, compatibilizer, thermal stability, bleaching, hybrid nanocomposites

## Abstract

The thermal, thermo-mechanical and flammability properties of kenaf core hybrid polymer nanocomposites reinforced with unbleached and bleached nanocrystalline cellulose (NCC) were studied. The studied chemical composition found that unbleached NCC (NCC-UB) had 90% more lignin content compared to bleached NCC (NCC-B). Nanocelluloses were incorporated within polypropylene (PP) as the matrix, together with kenaf core as a main reinforcement and maleic anhydride grafted polypropylene (MAPP) as a coupling agent via a melt mixing compounding process. The result showed that the thermal stability of the nanocomposites was generally affected by the presence of lignin in NCC-UB and sulfate group on the surface of NCC-B. The residual lignin in NCC-UB appeared to overcome the poor thermal stability of the composites that was caused by sulfation during the hydrolysis process. The lignin helped to promote the late degradation of the nanocomposites, with the melting temperature occurring at a relatively higher temperature of 219.1 °C for PP/NCC-UB, compared to 185.9 °C for PP/NCC-B. Between the two types of nanocomposites, PP/NCC-B had notably lower thermo-mechanical properties, which can be attributed to the poor bonding and dispersion properties of the NCC-B in the nanocomposites blend. The PP/NCC-UB showed better thermal properties due to the effect of residual lignin, which acted as a compatibilizer between NCC-UB and polymer matrix, thus improved the bonding properties. The residual lignin in PP/NCC-UB helped to promote char formation and slowed down the burning process, thus increasing the flame resistance of the nanocomposites. Overall, the residual lignin on the surface of NCC-UB appeared to aid better stability on the thermal and flammability properties of the nanocomposites.

## 1. Introduction

Recently, natural fibers applications are expected to become increasingly prevalent, mainly due to the increment of environmental issues, which entail the reduction in the applications of petroleum-based products [1,2,3]. Used petroleum-based polymers require hundreds of years to fully decompose [4,5]. This problem led to continuous global demand on improving living standards, which create the new development of technology based on renewable products [6]. Thus, natural fibers’ substitution as reinforcement over synthetic fibers in composite materials were seen to help in promoting environmental sustainability [7,8,9,10]. These so-called biocomposites can be easily decomposed due to their biodegradability properties [11,12,13,14,15,16]. Besides, the natural fibers were chosen due to its advantages such as being more economical, as well as environmentally friendly with comparably good performance in mechanical properties, making them the most recommended substitution material over glass fiber [17,18,19,20]. The other advantages include low density, renewability, biodegradability, non-toxicity, good insulation property and low machine wear [13,21]. However, the common disadvantages of natural fibers are their hydrophilic nature, which leads to lower interfacial bonding and surface adhesion with the matrix, which has been reported by many researchers [22,23,24,25,26,27,28]. This behavior leads to the deterioration of the properties of the biocomposites. Therefore, chemical modification to improve the surface properties of the fibers and matrix are seen to be necessary. Apart from the success of natural fibers to enhance the mechanical properties of the composites, the production of new natural fiber-based materials has opened up a strong opportunity, especially in the industrial sector. Nanocellulose is one of the products extracted from natural fibers, which possesses high performance with tailored mechanical and physical properties, making it the most attractive renewable material for advanced applications [29]. Nanocellulose is generally referring to cellulosic extracts with a diameter of <100 nm [30,31,32,33]. Meanwhile, cellulose is the main constituent in the lignocellulosic fibers, which is structurally organized into fibrils and surrounded by a matrix of lignin and hemicellulose [34,35,36]. The extraction of cellulose produces different types of nanocellulose: (i) nanocrystalline cellulose (NCC) [37]; and (ii) cellulose nanofibrils (NFC) [38]. NCC contains near-perfect crystallinity, which is usually produced via an acid hydrolysis process under controlled time and temperature [39]. To achieve a perfect nano-whisker-like NCC, the isolation of cellulose from lignin and hemicellulose usually has to take place; this is to ensure that the hydrolysis process was not hindered by the existence of amorphous materials.

Lignin, on the other hand, is one of the main components of cell walls that constituted in the lignocellulosic materials. Lignin is a complex chemical compound, a cross-linked polymer which gives strength to wood. It is formed by photosynthesis and represented approximately around 10–35% of lignocellulosic biomass [30,40,41]. In general, during the production of nanocellulose, lignin is usually delignified via the pulping process and multistage bleaching process to separate it from the fiber completely. Similar to the production of paper, the elimination of lignin and other amorphous component is crucial. In 1998, the papermaking industry contributed to massive isolated lignin by-product production, in which 99% were either burned or disposed of in the water stream. Since then, researchers have to focus on a large number of studies on utilizing waste lignin other than being used as fuel [40,41]. According to Rosa et al. [35], recent investigation has shown that the lignin can impart a positive effect on the thermal and mechanical properties as it blends in composites. According to Chen et al. [40], lignin has interesting properties, such as being renewable, non-toxic and biodegradable, which validate it into a value-added product. Furthermore, lignin could play a role as a dispersant to improve the dispersion of cellulose whiskers in the composite’s constituents [35,42,43]. Lignin can also withstand high-temperature degradation from 100 °C up to 900 °C, as lignin comprises aromatic rings with various branches, and the activity of chemical bond in lignin covered an extremely wide range [42]. In addition to other polymers such as bio-based polyurethane [43] and bio-epoxy [44] used as a flame retardant in composites, lignin was also seen as potential candidate for flame retardant materials. The aromatic chemical structure in lignin also stimulates the formation of a high char yield after decomposition, which can help protect the underlying substrate from further decomposition and improve flame retardancy [45,46].

According to Zheng, Li and Ek [45], lignin was studied as a flame retardant in plastics, namely polypropylene (PP), which produced a protecting layer and enhanced its fire retardancy. Besides that, a lignin-containing cellulose nanofibril (LCNF)-reinforced starch film was prepared by Zhang et al. [47]. The result showed that the LCNF increased the water barrier, thermal and mechanical properties of the starch film. Another study conducted by Yetiş et al. [48] on the acetylation of lignin-containing microfibrillated cellulose (Ac-MFLC) reinforced polylactic acid showed that Ac-MFLC significantly improved the crystallization rate of PLA. Bionanocomposites exhibited superior mechanical performance and thermal properties. Following this trend, Ballner et al. [49] utilized polystyrene with LCNF, followed by hot-pressing and obtained bionanocomposites with increased Charpy impact bending strength, compared to neat polystyrene. On the other hand, Nair et al. [50] investigated the effect of lignin on the mechanical, thermal and barrier properties of cellulose nanofibril-reinforced epoxy composite. The yielded bionanocomposites displayed high barrier, thermal and mechanical properties. The tensile strength of LCNF-reinforced epoxy composites increased from 65 MPa to 134 MPa, with the reinforcement of 23% of lignin content. This enhancement might be attributed to the improved compatibility between the LCNF and the epoxy matrix caused by the presence of residual lignin.

As the residual lignin in NCC might provide some advantages to the properties of the nanocomposite, it is worth investigating whether the complete bleaching of NCC is necessary. Based on a literature survey, none of the previous reports have dealt with the effects of the bleached and unbleached NCC on the thermal and flammability properties of polypropylene (PP) reinforced kenaf core hybrid polymer bionanocomposites. Therefore, in this study, the improvement of kenaf core reinforced polymer composites were observed by hybridizing the composites with NCC-UB and NCC generated from kenaf core. The NCC produced from kenaf core varied in the lignin content by controlling its bleaching process. The bleached and unbleached NCC were predicted to have a different lignin content and effect on the properties of kenaf core hybrid polymer nanocomposites, especially on the thermal, thermo-mechanical and flammability properties, and these were investigated.

## 2. Materials and Method

### 2.1. Materials

PP resin was supplied by Titan Petcham Sdn. Bhd. (Pasir Gudang, Johor, Malaysia), and maleic-anhydride grafted polypropylene (MAPP) was provided by Sigma Aldrich, Darmstadt, Germany. All the chemicals, namely sodium hydroxide (NaOH), anthraquinone (AQ), hydrogen peroxide (H_2_O_2_), sodium chlorite (NaClO_2_), acid acetic (CH_3_CO_2_H), acetone and ethanol used for the pre-treatment process, were provided by Sigma Aldrich (Darmstadt, Germany) and SYSTERM (Shah Alam, Selangor, Malaysia). In this study, for hybridization purposes, two types of fiber sizes were used, namely kenaf core and Kenaf NCC. Kenaf core was supplied in powder form by the National Kenaf and Tobacco Board (LKTN), Kelantan, Malaysia. NCC extracted from the kenaf core was obtained from the Malaysian Palm Oil Board (MPOB), Kajang Selangor, Malaysia.

### 2.2. Pulping of Kenaf

200 g of kenaf core chips with a size of 2–3 cm long were placed in a twin digester with 0.1% anthraquinone (AQ) and 20% sodium hydroxide (NaOH). The ratio of the kenaf core chips cooking liquid was set to 1:10, to make sure the fibers were completely soaked during the cooking process. The cooking process was carried out at a temperature of 170 °C for 90 min.

NaOH—AQ pulping was chosen for this method to reduce the rate of degradation and cause less damage to the cellulose chain. The AQ addition helps to impart a stabilization effect on the cellulose macromolecules, and is able to block the active group of cellulose and decrease the rate of oxidation [51].

### 2.3. Bleaching

The kenaf pulp was later purified through three-stage bleaching. Table 1 shows the bleaching conditions employed in the study. Two types of pulp were prepared: (1) Unbleached pulps, denoted as UB; and (2) pulps that were subjected to all bleaching stages (D_1_, followed by E_P_, then D_2_) and indicated as B2.

### 2.4. Isolation of Unbleached and Bleached Nanocellulose

**Figure 1:**
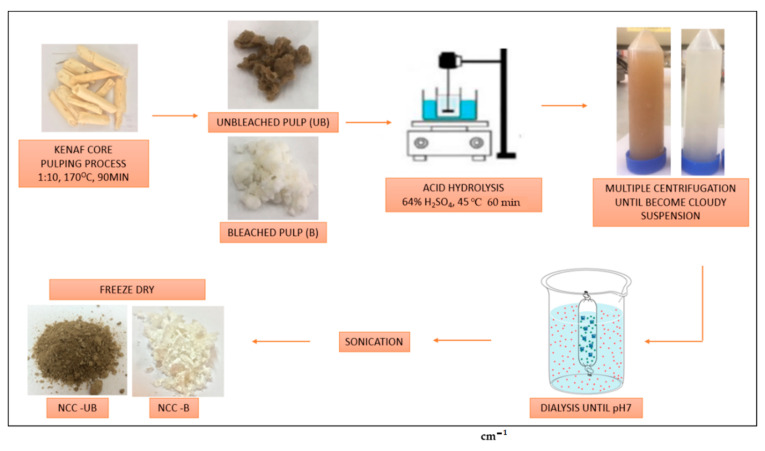
The schematic diagram of unbleached and bleached NCC isolation.

The unbleached and bleached pulps were proceeded with acid hydrolysis using 64% of sulfuric acid (H_2_SO_4_) for 60 min at 45 °C. Later, both unbleached and bleached pulps were rinsed using multiple centrifugations until the solution became a cloudy suspension. The process was continued with dialysis using cellulose membrane until the pH changed from acidic to neutral. The suspensions were then sonicated for 30 min before they were freeze-dried. The kenaf core NCC powder was kept in a cool and dry place for further experiments. The unbleached and bleached NCC were denoted as NCC-UB and NCC-B, respectively. Table 2 shows the acid hydrolysis conditions employed in the study, whilst the schematic diagram of unbleached and bleached NCC was depicted in Figure 1.

### 2.5. Fabrication of Kenaf Core Nanocellulose Hybrid Composites

Kenaf core nanocellulose hybrid composites were fabricated by incorporating two types of filler, namely NCC-UB and NCC-B. Those two fillers were incorporated within PP as the matrix and kenaf core as main reinforcement and MAPP as a coupling agent, which were set to constant value of 40 wt. % and 1 wt. %, respectively. All these materials were prepared via a melt mixing compounding process. The nanocomposites were mixed using HAAKE Polydrive R600 (Massachusetts, United States), Germany internal mixer at constant temperature and speed of 180 °C and 50 rpm, respectively. The compounded samples were then crushed into small pieces using a crusher. The samples were then pressed into the specific mold of 200 mm × 200 mm × 3 mm using a VECHNO VATION (Indiana, United Sates), a 40-tonne hot press machine, at 180 °C for 8 min (3 min for melting to take place and 5 min for curing). The compositions of each nanocomposite are listed in Table 3.

### 2.6. Characterization of Kenaf Core Nanocellulose

#### 2.6.1. Lignin Composition of Bleached and Unbleached Kenaf Core

The insoluble lignin in kenaf core pulps was evaluated according to TAPPI T222 om-02, whilst the kappa number was determined according to TAPPI 236 om-99 standard.

#### 2.6.2. Morphological Analysis

The images of nanocrystalline cellulose from kenaf core were observed via TEM machine brand JEOL JEM-2100, Tokyo, Japan. The TEM machine utilized an energetic electron to provide morphologic, compositional and crystallographic information on the sample at a maximum potential magnification of 1 nm. To have an enhancement in contrast, especially for an ultra-thin sample like NCC, the samples were prepared via a post-staining technique using uranyl acetate.

### 2.7. Characterization of Kenaf Core Nanocellulose Hybrid Composites

#### 2.7.1. Thermogravimetric Analysis (TGA)

The thermal composition and thermal stability of the NCC at different lignin content were done using a TGA/SDTA 851 brand Mettler Toledo (Ohio, United States) thermogravimetric analyzer. The tested temperature range was from 50–700 °C, with a heating rate of 10 °C/min under nitrogen atmosphere.

#### 2.7.2. Differential Scanning Calorimetry (DSC)

The DSC test was performed using Perkin Elmer Instrument (Massachusetts, United States). The temperature range was from 50–300 °C with a heating rate of 10 °C/min. The fractional crystallinity was calculated according to Equation (1)

*X_c_* = (∆*Hf*)/(∆*Hfo W*) × 100 (1)

where ∆*Hf* is the heat of fusion of the sample, ∆*Hfo* is the heat of fusion of 100% crystalline PP = 207 J/g, and W is the mass fraction of PP in the composites.

#### 2.7.3. Dynamic Mechanical Analysis (DMA)

The DMA test was carried out using Perkin Elmer DMA (Massachusetts, United States) with a temperature range of −50 to 150 °C. The heating rate was set to10 °C/min, and the frequency was 1 Hz. In this test, the ASTM D7028 standard was followed, and the dimension of the sample was 3 mm × 127 mm × 63 mm.

#### 2.7.4. Flammability Testing

The flammability test was carried out according to UL94 Horizontal Burning testing using ASTM 635 standard to determine the tendency of the sample to extinguish or spread the flame once the sample was ignited. Sample with 3 mm × 127 mm × 63 mm dimension was subjected to flame tilted at 45° for 30 s, or until it reached the first mark. The sample was left to be burned after the removal of the flame up to the second mark. The burning time between the first and second mark was recorded, and the burning rate was calculated according to Equation (2)

Horizontal Burning Rate (mm/sec) = 60 L/t(2)

where L = length of the sample burnt within the 75 mm range of the sample, while t = time taken in seconds for the sample to burn within a 75 mm range of the sample.

## 3. Results and Discussion

### 3.1. Chemical and Morphological Properties of Unbleached and Bleached Kenaf Core NCC

The lignin content and kappa number of raw kenaf core, unbleached and bleached kenaf core are listed in Table 4. As expected, the raw kenaf core showed the highest lignin content, as the fiber was not subjected to any chemical treatment. The amount of lignin in raw kenaf core was 33.7%, which was aligned with the findings reported by Hatakeyama and Hatakeyama [52], who reported that the amount of lignin of woody materials varied from 12% to 39%. The percentage of lignin was significantly decreased for unbleached kenaf core. The decrement of lignin content might be attributed to the pulping process done upon kenaf core pulp, in which a major amount of lignin, wax and oil covering the fiber cell and hemicellulose were removed [22,23,24]. The amount of lignin for unbleached kenaf core was further decreased to 0.3% after the completion of the bleaching process (bleached kenaf core). The value of the kappa number was observed to correlate well with the lignin content, where it decreased with decreasing lignin content.

**Figure 2:**
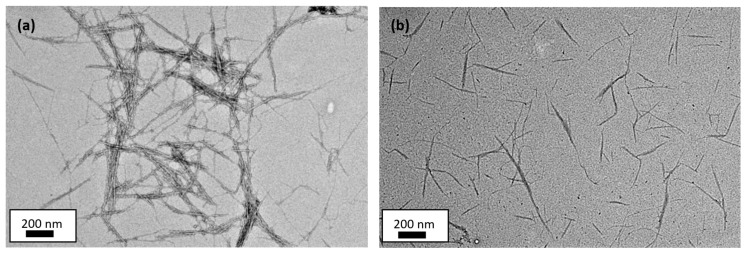
The TEM images of (**a**) unbleached NCC (NCC-UB) and (**b**) bleached kenaf core NCC (NCC-B).

The kenaf core pulps were then subjected to an acid hydrolysis process to yield two types of NCC, and the images of both NCC were depicted in Figure 2. The TEM images of the unbleached and bleached kenaf core NCC were obtained on a 200 nm scale. Generally, both unbleached and bleached NCCs showed a needle-like or rod-like structure with a diameter of ~37 nm and ~70 nm, respectively. According to Sharma et al. [30], Zainuddin et al. [53] and Brinchi et al. [54], the diameter of NCC usually falls within the range of 5–70 nm. Both NCC-UB and NCC-B in this study fell within this range. In addition, the length of the obtained NCC-UB was observed to appear averagely longer, in the vicinity of 400–600 nm, compared to that of NCC-B with the value of 200–300 nm. Different extent of dispersion was observed from both Figure 2a,b, where unbleached NCC (NCC-UB) showed lower dispersion, while bleached NCC (NCC-B) was seen to disperse evenly. The findings could be attributed to the fact that most of the NCC-B had individual fibrils resulting from the complete bleaching process. On the contrary, nanowhiskers in NCC-UB were still intact due to the presence of lignin component, which resulted from the absence of the bleaching process. High lignin content was known to hinder the defibrillation of the fiber, and thus lead to the formation of nanocellulose bundles, as shown in Figure 2a [55].

### 3.2. Thermal Properties of Kenaf Core Hybrid Nanocomposites

#### 3.2.1. Thermogravimetric Analysis (TGA)

**Figure 3:**
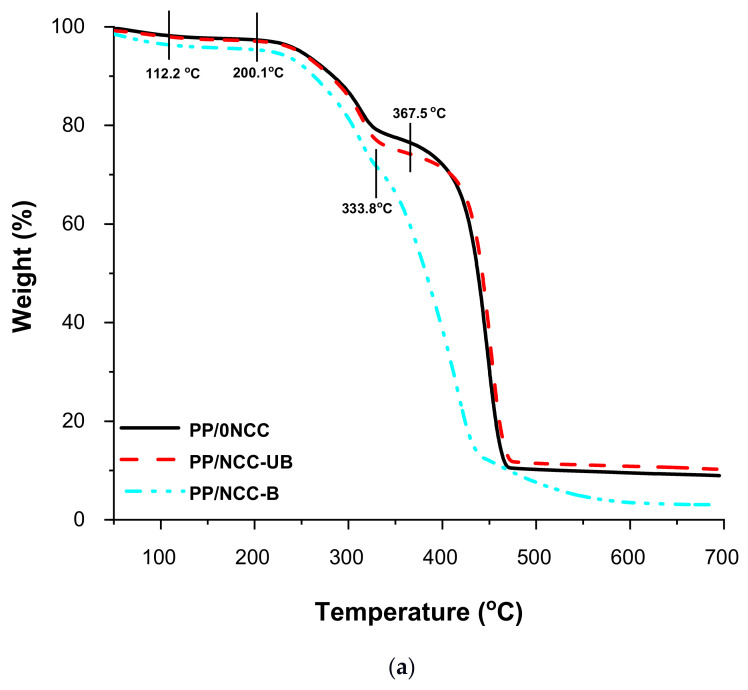

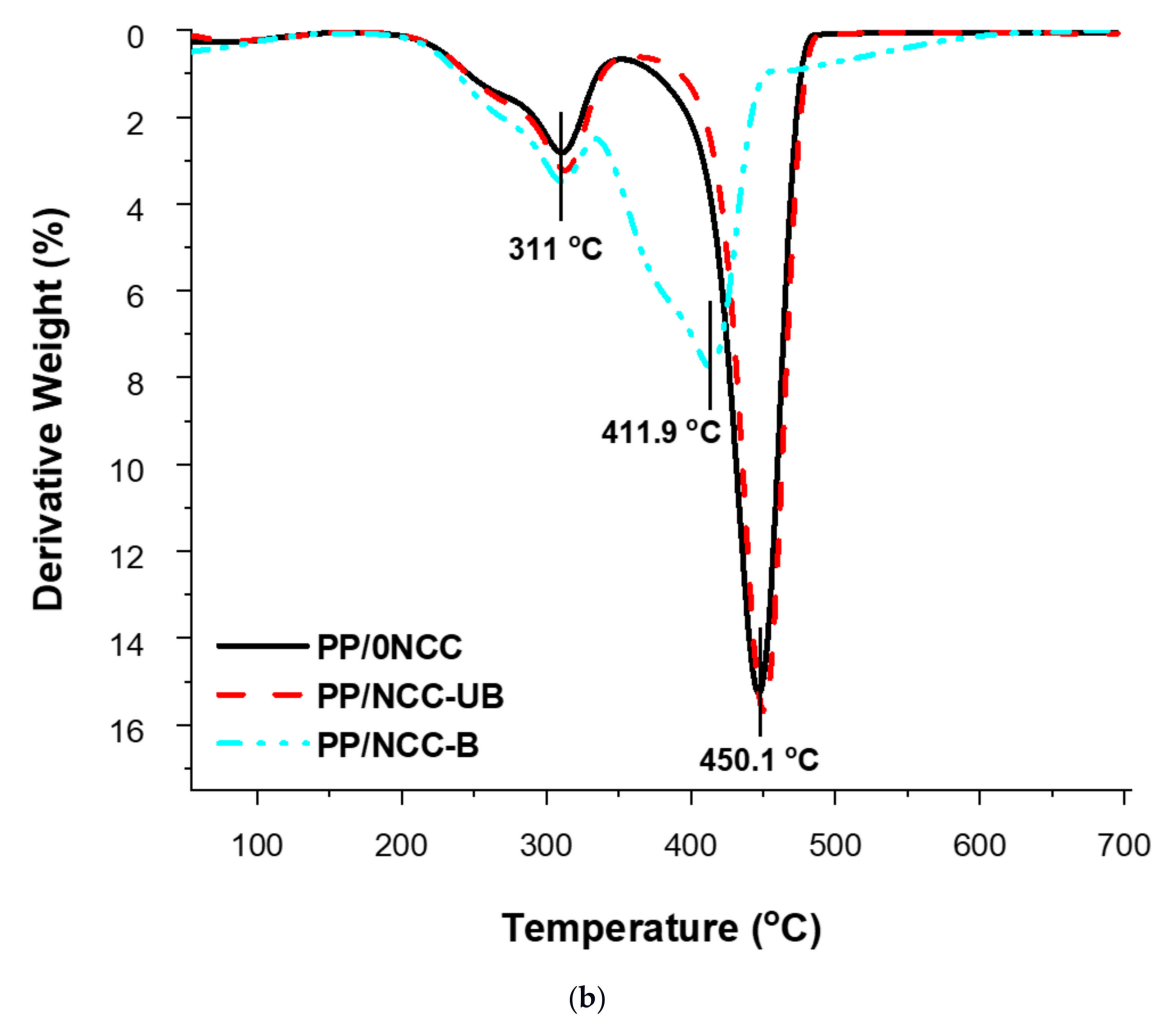
The (**a**) thermogravimetric analysis (TGA); and (**b**) DTG graphs for kenaf core nanocomposites reinforced bleached and unbleached NCC.

The thermal degradation curves of kenaf core hybrid nanocomposites, with the addition of unbleached and bleached NCC, were depicted in Figure 3 and Table 5. The degradation of the nanocomposites usually occurs in multiple stages, comprising the evaporation of water at the first stage, followed by the decomposition of the nanocomposites [56]. The early thermal degradation stage in this study occurred at 100–111.2 °C, which contributed to 5% of the total weight loss. This phenomenon corresponded to the evaporation of water molecules [13,28,29,30]. It includes three types of water in fiber, namely free water, loosely-bonded water and chemically-bonded water, which were lost due to the overlapping of three different processes, as recorded by the thermogravimetric curve, as shown in Figure 3a [57]. The second degradation of the nanocomposites was started in the range of 198.9–200.1 °C. It was attributed to the degradation of the lignocellulosic component, mainly hemicellulose in kenaf core, and some that remained in unbleached NCC. Hemicellulose can be easily decomposed at a temperature between 220–315 °C due to its random and amorphous structure, which were very easy to remove from the main stem and degrade at low temperatures [58]. The final degradation that was initiated at around 333.8–367.5 °C was contributed by the cellulose degradation in both kenaf core and NCC. Unlike hemicellulose, cellulose comprises long polymer glucose, good structure order and is very strong, and hence has high thermal stability.

Apart from the above findings, the types of NCC added in the kenaf core composites did show variation in the thermal degradation curves. Generally, the degradation temperature of the composites was shifted to a lower temperature once added with NCC. This can be related to the unstable sulfate groups that existed from the extracted nanocellulose after the sulfuric acid hydrolysis process [59]. According to Gan et al. [56], the presence of the sulfated group on the nanocellulose outer surface was caused by the replacement of the hydroxyl group with the sulfated group during the hydrolysis process. The sulfated group acted as a catalyst in degrading the nanocellulose in facilitating the breakdown of nanocellulose when the temperature reached 200 °C, thus lowering its thermal stability. From the observation of TGA and DTG curves in Figure 3, PP/NCC-B showed a significant impact with lower degradation temperature compared to PP/0NCC and PP/NCC-UB. The DTG curve of PP/NCC-B showed that the main degradation of the nanocomposites occurred at a lower temperature in the vicinity of 411.9 °C, whilst the degradation of both PP/0NCC and PP/NCC-UB took place at a higher temperature, which was around 450.1 °C.

The above results can be attributed to the effect of the chemical composition of the NCC. The NCC-B contained almost 99% cellulose. This might be due to the effect of sulfation during the hydrolysis process. The higher the sulfation area, the more accessible the cellulose to degradation that lowers the decomposition temperature [56]. On the other hand, unbleached NCC (NCC-UB) contained almost 33% lignin, which assisted in hindering the replacement of the hydroxyl group with sulfate groups, thus lowering the sulfation effect. Furthermore, lignin can also protect the NCC from early decomposition, as reported by Yang et al. [42]; lignin was the most difficult substance to decompose, as it happened slowly under ambient temperature to 900 °C. The results suggested that the remaining lignin in NCC-UB helped to neutralize the sulfation effect imparted from the sulfate groups as a by-product of the acid hydrolysis process, thus sustaining the thermal stability of the nanocomposites. This effect was also attributed to the higher remaining residue of PP/NCC-UB (~16%), which was slightly higher than PP/0NCC (~15%) up to 900 °C. The remaining residue of PP/NCC-B, however, was observed to be the lowest, at around 5%, caused by the high degradation activities due to the sulfation effect.

#### 3.2.2. Differential Scanning Calorimetry (DSC)

**Figure 4:**
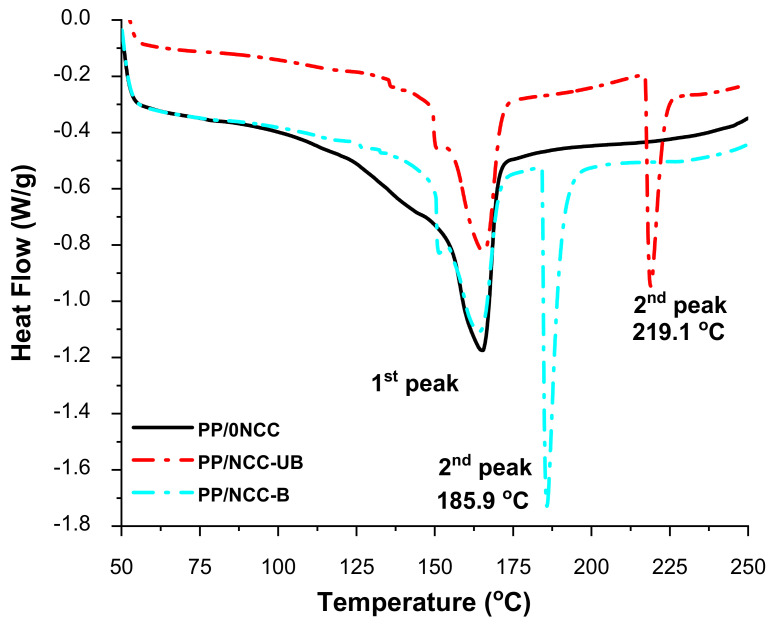
The DSC graph of kenaf core nanocomposites reinforced bleached and unbleached NCC.

The DSC curves of the nanocomposites are presented in Figure 4, and information on the DSC analysis was listed in Table 6. As observed from the graph in Figure 4, all samples shared comparable values at the first endothermic peaks at around 165 °C. According to Phiri, Khoathane and Sadiku [60], the melting peak of polymer PP occurred at around 160 °C, and was increased to 162 °C when added with 15 and 30% of natural fiber filler. The PP/0NCC showed a slightly higher value at 165 °C, which might correspond to the higher addition of kenaf core filler (40%), which increased the interaction between matrix and filler, and led to a restriction in the polymer chain of the composites. The value was seen to be comparable with nanocomposites added with unbleached NCC (PP/NCC-UB), while the value was observed to slightly decrease when the composite was added with bleached NCC (PP/NCC-B). A more noticeable effect on the type of NCC in the nanocomposites on its thermal properties can be observed through the second peaks of the DSC curves. PP/NCC-UB peaks occurred at a later temperature around 219 °C compared to PP/NCC-B, which took place at 185 °C. These results were in good agreement with the above discussion, where the effect of the sulfate group on the surface of NCC due to the post effect of acid hydrolysis process decreased the nanocomposites’ thermal stability, especially the one added with NCC-B [35,56].

Also included in Table 6 are the value of enthalpy of fusion, ∆*H_f_* and the value of fractional crystallinity of the composites with and without NCC loading. Both values were observed to decrease for samples with added NCC. It was suggested that the addition of NCC impeded the movement of the molecular chain of the composites, thus reducing the regular arrangement. The presence of NCC caused the molecular chain to be less likely to diffuse into the crystal nucleus, and lowered the crystal growth rate that subsequently decreased the crystallinity [61]. Instead of being the sufficient nucleating agent, the surface of NCC was partially attached to the sulfate group as a by-product of the acid hydrolysis process, and limited the nucleation of the PP matrix, which decreased the crystallinity of the nanocomposites.

#### 3.2.3. Dynamic Mechanical Analysis (DMA)

**Figure 5:**
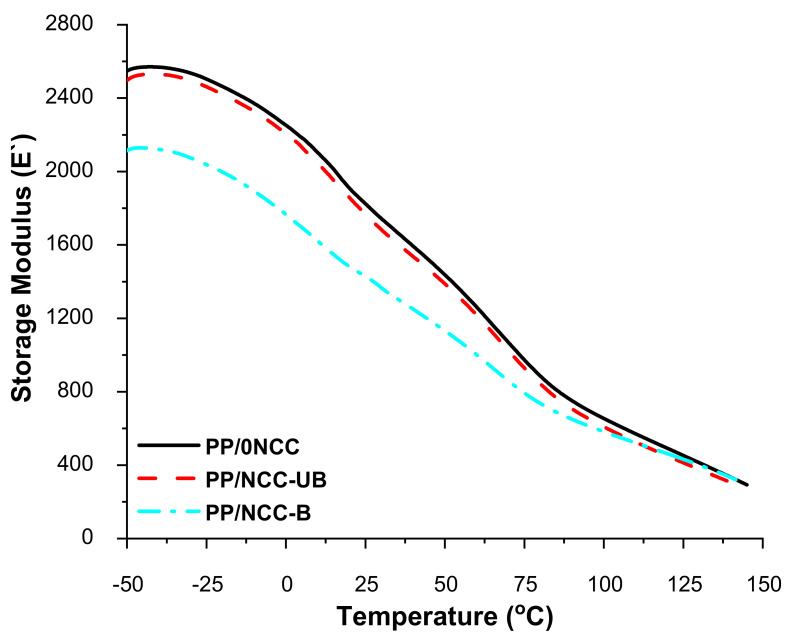
The storage modulus of kenaf core nanocomposites reinforced bleached and unbleached NCC.

The thermomechanical properties of the kenaf core hybrid nanocomposites were determined via DMA. The storage modulus or dynamic modulus is typically related to Young’s Modulus, which is also associated with the stiffness of a material. Generally, from the storage modulus E′ value of the nanocomposites shown in Figure 5, one can see that the curves for all samples were broader at the low temperature, and decreased as the temperature increased. This occurrence represented the orientation of the component, where it was tightly packed at the frozen state, and became loose and increased in mobility as the temperature was increased [62].

The high storage modulus of PP/0NCC was attributed to the high stiffness or toughness properties of PP/0NCC, which imparted from the addition of 1 wt.% of the MAPP coupling agent, which helped enhance the compatibility and surface adhesion between the polymer matrix and fiber filler component of the sample [35,37]. Meanwhile, the addition of NCC-UB in the nanocomposites did not significantly impact the storage modulus properties. On the other hand, the addition of NCC-B markedly decreased the intensity of the modulus, especially at temperatures lower than 100 °C.

**Figure 6:**
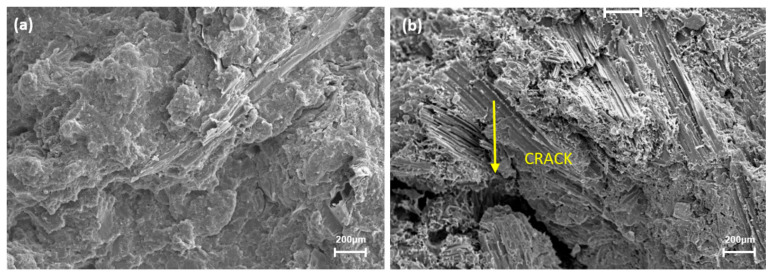
The morphology properties of the flexural fracture surface of: (**a**) PP/NCC-UB; and (**b**) PP/NCC-B.

The occurrence might be associated with poor NCC dispersion and agglomeration in the polymer, and with the addition of 1 wt.% of the MAPP coupling, this was insufficient to help to improve the bonding properties. According to Gan et al. [56], low nanocellulose loading did not show any changes to the dynamic mechanical properties of nanocomposites. Therefore, higher loadings are needed to improve the properties. However, the storage modulus of nanocomposites added with NCC-B was the lowest compared to that PP/NCC-UB. This was caused by the agglomeration of high crystalline NCC-B that imparted brittleness properties, thus lowering the storage modulus. This occurrence was supported by the images of the fracture surface on the flexural tested sample for both PP/NCC-UB and PP/NCC-B in Figure 6. The morphological properties of PP/NCC-UB showed a very clear surface, with no sign of cracks otherwise. The formation of cracks can be attributed to the high brittleness explained by the low storage modulus value of the sample.

**Figure 7:**
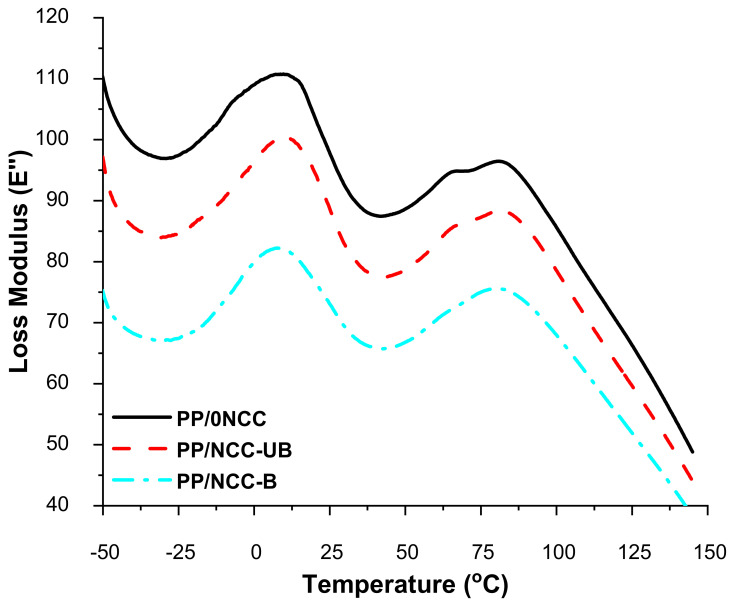
The loss modulus of kenaf core nanocomposites for reinforced bleached and unbleached NCC.

The loss modulus is known as the viscous response of material over temperature, and the results are depicted in Figure 7. The loss modulus of PP/0NCC showed the highest value, indicating higher viscosity due to superior molecular bonding between the PP matrix and kenaf core filler, with the help of 1 wt.% of MAPP, which increased the stiffness and imposed restriction of molecular movement [38,39]. The addition of NCC was supposed to improve the rigidity of the nanocomposites and enhance the strength; however, the addition of NCC-UB and NCC-B showed the opposite results. These results might be attributed to the NCCs’ agglomeration, and increased the mobility of the molecules in the nanocomposites component. The addition of NCC-UB in the PP/NCC-UB nanocomposites, on the other hand, showed a better loss of modulus value compared to PP/NCC-B. This observation might be associated with the residual lignin on the surface of the NCC-UB that helped to give a compatibilizing effect and impart better adhesion between the NCC and PP matrix. This finding was in line with Rozman et al. [63], who stated that lignin contains both polar hydroxyl group and non-polar hydrocarbon and benzene rings, which play a vital role in enhancing the compatibility between the NCC and PP matrix.

The graphs also showed the appearance of two major peaks at around 10 °C and 80 °C. The peak at 10 °C corresponded to the β-transition, representing the glass rubber transition attributed to molecular motion related to the unrestricted amorphous PP, which can be affected by the degree of particle dispersion [64]. Sample PP/NCC-UB and PP/NCC-B showed a lower loss modulus peak at 10 °C, compared to the PP/0NCC sample, which reflected its degree of particle dispersion in nanocomposites.

**Figure 8:**
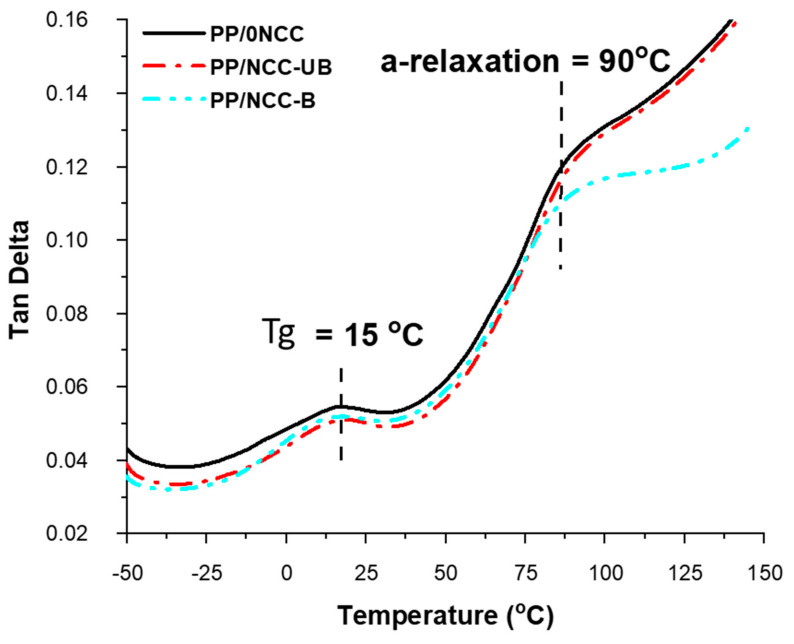
The tan delta graph of kenaf core nanocomposites for reinforced bleached and unbleached NCC.

The tan delta (tan δ) of the kenaf core polymer nanocomposites were depicted in Figure 8. Guo et al. [65] reported in their study that the tan δ curve of PP represented three relaxations located at around −80 °C (γ), 10 °C (β) and 100 °C (α). Most studies usually focused on the finding on β-relaxation of PP, which corresponded to the glass rubber transition of the amorphous portion and the temperature at the maximum peak assigned to the glass transition temperature (Tg). In this study, all samples shared the same value of Tg at 15 °C, which was higher than the Tg of PP, indicating that kenaf core fiber and NCC addition helped to confine the movement of the macromolecular segment [66]. Meanwhile, the second higher peak of tan δ at 90 °C in Figure 8 corresponded to α-transition related to PP crystalline fractions [13,41].

Other than that, tan δ also provides information on the damping properties of the samples. As observed from Figure 8, all samples showed a comparable value of tan δ, except after the α-relaxation temperature. The tan δ values of PP/NCC-B showed a marked decrement after 90 °C; an indication of low damping value reflecting the agglomeration effect of NCC-B in the nanocomposites and imparting high stiffness, as well as restricting the chain mobility [67].

### 3.3. Flammability Properties of Kenaf Core Hybrid Nanocomposites

**Figure 9:**
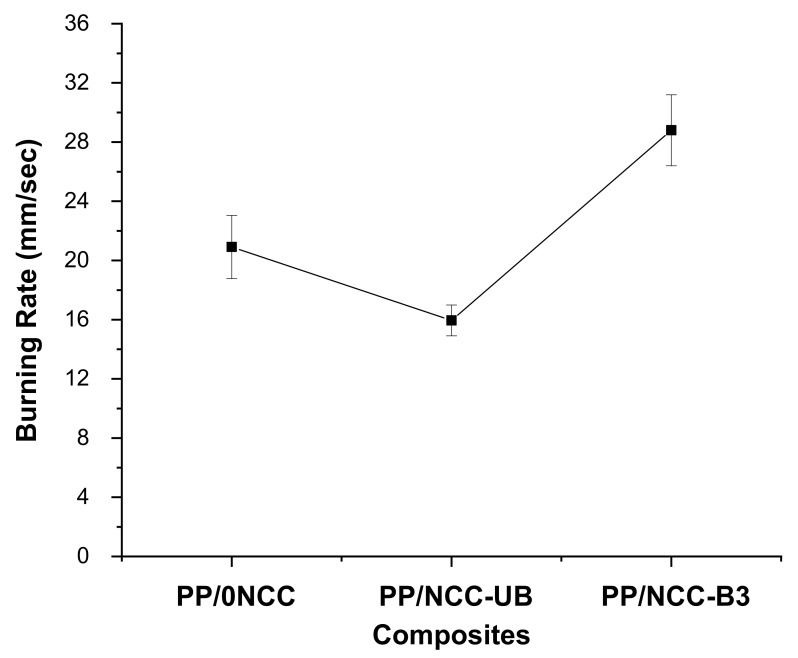
The burning rate of kenaf core nanocomposites for reinforced bleached and unbleached NCC.

**Figure 10:**
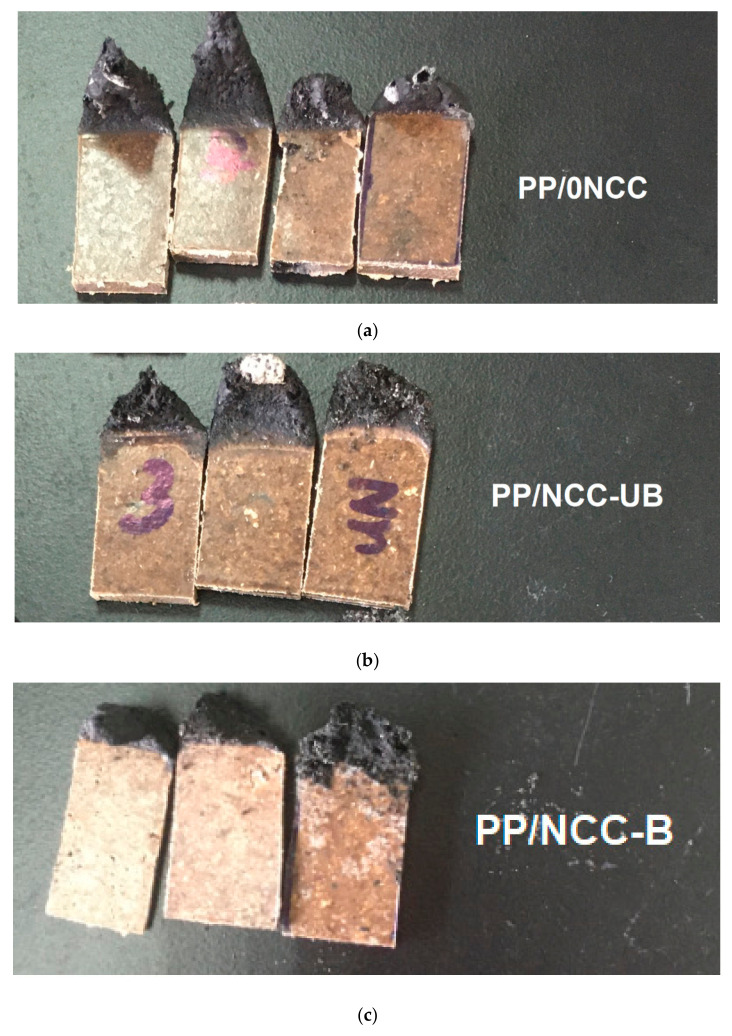
The images of (**a**) PP/0NCC, (**b**) PP/NCC-UB and (**c**) PP/NCC-B.

The flammability test of the kenaf core hybrid nanocomposites was carried out via the UL94 horizontal burning test (UL94-HB). The burning rate of the samples was depicted in Figure 9, where the low burning rate indicated better flame retardancy properties. The mixed trend of the burning rate was observed for all samples. Despite different burning rates recorded, all samples were observed to be accompanied by a flame or glow, and formed drip of samples as they burned, as shown in Figure 10 for PP/0NCC (Figure 10a), unbleached NCC (NCC-UB) (Figure 10b) and bleached kenaf core NCC (NCC-B) (Figure 10c) after a flammability test.

From the graph in Figure 9, the burning rate was observed to drop from 20.90 ± 2.14 mm/sec to 15.95 ± 1.04 mm/sec when kenaf core composites (PP/0NCC) were added to unbleached NCC (PP/NCC-UB). In contrast, the sample with bleached NCC (PP/NCC-B) was seen to have the highest burning rate, with a value of 28.79 ± 2.40 mm/sec, indicating lower flame retardancy. The burning properties of the composites with the addition of natural fibers can be affected by the amount of cellulosic materials added in the systems. Natural fibers are expected to act as combustion sources in composites. However, with the presence of coupling agent that helps to increase the bonding and promote a more uniform and homogenous blend of fibers in the polymer matrix, restricting the flow-ability of PP and reducing the dripping effect of the composites, fire retardancy of the samples could be improved [42,43]. According to Subasinghe, Das and Bhattacharyya [68], kenaf composites formed a more stable layer of char in a fully burnt state. Good distribution of kenaf fibers helped to create a barrier between burnt and unburnt materials and inhibited the fire growth, as well as reducing volatiles and oxygen content present in the fire boundary.

The burning rate was decreased once added with unbleached NCC (NCC-UB), and the reduction was attributed to the residual lignin that existed on NCC surface. The NCC-UB contained 11.5% of lignin content with a kappa number of 32.2; more than 90% higher than that of bleached NCC (NCC-B). Although the amount of lignin for the whole nanocomposites system was considered to be very small, the high surface area possessed by NCC helped to impart the effect of lignin to the nanocomposites. Lignin was known to have an aromatic chemical structure that forms char after the decomposition [19,44]. The formation of char is crucial in gauging the flammability properties [69]. The formation of the char layer can help to improve flame retardancy by protecting the underlying substrate for further decomposition and slowing down the burning rate.

On the contrary, the burning rate of the PP/NCC-B was observed to have the highest value compared to PP/0NCC and PP/NCC-UB. The failure related to the homogeneity of the filler in the polymer matrix and the existence of sulphate by-products grafted on the NCC surface might explain the unsatisfactory properties. The difference in the surface energy of nanocellulose, together with their intrinsic tendency to agglomeration, led to poor interfacial adhesion and inhomogeneous dispersion of nanocellulose, thus reducing its properties [58]. It is suggested that only 1 wt.% of MAPP in the system was incapable of improving the bonding between NCC-B and polymer matrixes. Meanwhile, the presence of the sulfate group affected NCC-B more severely compared to NCC-UB, which might be due to the existence of lignin. The sulfate group might act as a catalyst to reduce its thermal stability, and also be responsible for decreasing its resistance to early ignition, thus increasing the burning rate properties [27,45].

## 4. Conclusions

Kenaf core hybrid polymer nanocomposites were prepared with the addition of bleached and unbleached NCC. The content of lignin of each NCC was analyzed during its production, and the unbleached NCC (NCC-UB) showed higher lignin content, which was more than 90% than that of bleached NCC (NCC-B). The thermal stability of the kenaf core hybrid nanocomposites was generally affected by the sulfation of NCC and the amount of lignin content in NCC. The results showed that the residual lignin, particularly in NCC-UB (11.5% lignin content), helped to overcome the replacement of the hydroxyl group with the sulfate group that hindered the early degradation and shifted the main degradation temperature to a higher temperature, from 411.9 °C to 450.1 °C. Meanwhile, the thermo-mechanical properties of nanocomposites showed no significant improvement after the addition of both NCC. However, PP/NCC-UB showed slightly better thermo-mechanical properties compared to PP/NCC-B, which was attributed to the effect of lignin that can act as a compatibilizing agent to improve the bonding between matrix and NCC. The existence of lignin in NCC-UB helps to improve the flammability properties of the nanocomposites. The flammability rate behavior of NCC-UB hybrid nanocomposites decreased by 28.79 m/s, compared to NCC nanocomposites, which was 15.95 m/s. The remaining lignin on the surface of NCC-UB helped to produce the char layer, and slowed down the burning process. Therefore, in conclusion, the residual lignin presence on the surface of NCC-UB did help to impart good thermal and flammability properties. Even though NCC-UB was also exposed to the sulfation effect as a product of the acid hydrolysis process, lignin helped to overcome the effect by preventing the sulfated group replacement on the surface of NCC. Lignin also assisted to provide a compatibilizing effect by slightly improving the bonding between the NCC-UB and polymer matrix, which aided to retain the thermo-mechanical properties close to the control sample.

## Figures and Tables

**Table 1:** Bleaching condition for bleached kenaf core pulp.

Bleaching Stage	Chemical Charge	Reaction Time (min)	Temperature (°C)	Consistency (%)
D_1_	2% Sodium Chlorite 3% Acetic Acid	120	70	10
E_P_	1.5% NaOH 1% H_2_O_2_	90	70	10
D_2_	1.5% Sodium Chlorite 3% Acetic Acid	90	60	10

**Table 2:** Acid hydrolysis conditions employed for the production of nanocrystalline cellulose (NCC) from kenaf core.

Sample	Acid Concentration (%)	Reaction Time (min)	Temperature (°C)
NCC-UB	64% H_2_SO_4_	60	45
NCC-B	64% H_2_SO_4_	60	45

**Table 3:** The composition and denotation of the kenaf core nanocomposites.

Sample	NCC	Matrix	Kenaf Core	MAPP
	wt %	wt %	wt %	wt %
PP/0NCC	0	59	40	1
PP/NCC-UB	1	58	40	1
PP/NCC-B	1	58	40	1

**Table 4:** The lignin composition and the particle size of unbleached and bleached NCC.

Sample	Lignin Content (%)	Kappa No
Raw KC	33.7	36.7
Unbleached KC (UB)	11.5	32.2
Bleached KC (B)	0.3	2.0

**Table 5:** The thermal properties for kenaf core nanocomposites reinforced bleached and unbleached NCC.

	PP/0NCC	PP/NCC-UB	PP/NCC-B
Onset 1st stage (°C)	102.3	111.2	101.4
Onset 2nd stage (°C)	199.2	200.1	198.9
2nd stage main degradation temperature (°C)	313.0	311.1	311.1
Onset 3rd stage (°C)	348.0	367.5	333.8
3rd stage main degradation temperature (°C)	447.2	450.1	411.9
Residue (%)	9.04	10.23	3.49

**Table 6:** The important differential scanning calorimetry (DSC) values of kenaf core nanocomposites reinforced bleached and unbleached NCC.

Sample	Enthalpy, ∆*H_f_* (J/g)	Crystallinity, *X* (%)	1st Endothermic Peak (°C)	2nd Endothermic Peak (°C)
PP/0NCC	87.14	71.35	165.05	-
PP/NCC-UB	71.07	59.19	165.62	219.13
PP/NCC-B	61.67	51.36	163.93	185.77

## References

[1] Ilyas R.A., Sapuan S.M. (2020). Biopolymers and Biocomposites: Chemistry and Technology. Curr. Anal. Chem..

[2] Ilyas R.A., Sapuan S.M. (2020). The Preparation Methods and Processing of Natural Fibre Bio-polymer Composites. Curr. Org. Synth..

[3] Mazani N., Sapuan S.M., Sanyang M.L., Atiqah A., Ilyas R.A. (2019). Design and Fabrication of a Shoe Shelf From Kenaf Fiber Reinforced Unsaturated Polyester Composites. Lignocellulose for Future Bioeconomy.

[4] Aisyah H.A., Paridah M.T., Sapuan S.M., Khalina A., Berkalp O.B., Lee S.H., Lee C.H., Nurazzi N.M., Ramli N., Wahab M.S. (2019). Thermal Properties of Woven Kenaf/Carbon Fibre-Reinforced Epoxy Hybrid Composite Panels. Int. J. Polym. Sci..

[5] Abral H., Atmajaya A., Mahardika M., Hafizulhaq F., Kadriadi, Handayani D., Sapuan S.M., Ilyas R.A. (2020). Effect of ultrasonication duration of polyvinyl alcohol (PVA) gel on characterizations of PVA film. J. Mater. Res. Technol..

[6] Trache D., Thakur V.K., Boukherroub R. (2020). Cellulose nanocrystals/graphene hybrids—a promising new class of materials for advanced applications. Nanomaterials.

[7] Syafiq R., Sapuan S.M., Zuhri M.Y.M., Ilyas R.A., Nazrin A., Sherwani S.F.K., Khalina A. (2020). Antimicrobial activities of starch-based biopolymers and biocomposites incorporated with plant essential oils: A review. Polymers (Basel).

[8] Atikah M.S.N., Ilyas R.A., Sapuan S.M., Ishak M.R., Zainudin E.S., Ibrahim R., Atiqah A., Ansari M.N.M., Jumaidin R. (2019). Degradation and physical properties of sugar palm starch/sugar palm nanofibrillated cellulose bionanocomposite. Polimery.

[9] Asyraf M.R.M., Ishak M.R., Sapuan S.M., Yidris N., Ilyas R.A. (2020). Woods and composites cantilever beam: A comprehensive review of experimental and numerical creep methodologies. J. Mater. Res. Technol..

[10] Azammi A.M.N., Ilyas R.A., Sapuan S.M., Ibrahim R., Atikah M.S.N., Asrofi M., Atiqah A. (2020). Characterization studies of biopolymeric matrix and cellulose fibres based composites related to functionalized fibre-matrix interface. Interfaces in Particle and Fibre Reinforced Composites.

[11] Lakshumu Naidu A., Jagadesh V., Raju Bahubalendruni M.V. (2017). A review on chemical and physical properties of natural fiber reinforced composites. Int. J. Adv. Res. Eng. Technol..

[12] Gurunathan T., Mohanty S., Nayak S.K. (2015). A review of the recent developments in biocomposites based on natural fibres and their application perspectives. Compos. Part. A Appl. Sci. Manuf..

[13] Nguong C.W., Lee S.N.B., Sujan D. (2013). A Review on Natural Fibre Reinforced Polymer Composites. Int. J. Mater. Metall. Eng..

[14] Ayu R.S., Khalina A., Harmaen A.S., Zaman K., Isma T., Liu Q., Ilyas R.A., Lee C.H. (2020). Characterization Study of Empty Fruit Bunch (EFB) Fibers Reinforcement in Poly(Butylene) Succinate (PBS)/Starch/Glycerol Composite Sheet. Polymers (Basel).

[15] Jumaidin R., Ilyas R.A., Saiful M., Hussin F., Mastura M.T. (2019). Water Transport and Physical Properties of Sugarcane Bagasse Fibre Reinforced Thermoplastic Potato Starch Biocomposite. J. Adv. Res. Fluid Mech. Therm. Sci..

[16] Jumaidin R., Saidi Z.A.S., Ilyas R.A., Ahmad M.N., Wahid M.K., Yaakob M.Y., Maidin N.A., Rahman M.H.A., Osman M.H. (2019). Characteristics of Cogon Grass Fibre Reinforced Thermoplastic Cassava Starch Biocomposite: Water Absorption and Physical Properties. J. Adv. Res. Fluid Mech. Therm. Sci..

[17] Nurazzi N.M., Khalina A., Sapuan S.M., Ilyas R.A., Rafiqah S.A., Hanafee Z.M. (2020). Thermal properties of treated sugar palm yarn/glass fiber reinforced unsaturated polyester hybrid composites. J. Mater. Res. Technol..

[18] Norizan M.N., Abdan K., Ilyas R.A., Biofibers S.P. (2020). Effect of fiber orientation and fiber loading on the mechanical and thermal properties of sugar palm yarn fiber reinforced unsaturated polyester resin composites. Polimery.

[19] Nurazzi N.M., Khalina A., Sapuan S.M., Ilyas R.A. (2019). Mechanical properties of sugar palm yarn/woven glass fiber reinforced unsaturated polyester composites: Effect of fiber loadings and alkaline treatment. Polimery.

[20] Pappu A., Pickering K.L., Thakur V.K. (2019). Manufacturing and characterization of sustainable hybrid composites using sisal and hemp fibres as reinforcement of poly (lactic acid) via injection moulding. Ind. Crop. Prod..

[21] Asyraf M.R.M., Rafidah M., Ishak M.R., Sapuan S.M., Ilyas R.A., Razman M.R. (2020). Integration of TRIZ, Morphological Chart and ANP method for development of FRP composite portable fire extinguisher. Polym. Compos..

[22] Gupta H., Kanaujia K.K., Abbas R.S.M., Shukla R. (2019). A review on the mechanical properties of Natural fibre reinforced Polypropylene Composites. Int. Res. J. Eng. Technol..

[23] Ramesh M., Nijanthan S. (2016). Mechanical property analysis of kenaf-glass fibre reinforced polymer composites using finite element analysis. Bull. Mater. Sci..

[24] Paridah M.T., Juliana A.H., Zaidon A., Abdul Khalil H.P.S. (2015). Nonwood-based composites. Curr. For. Rep..

[25] Julkapli N.M., Bagheri S., Sapuan S.M., Salit M.S. (2015). Manufacturing of Natural Fibre Reinforced Polymer Composites. Manufacturing of Natural Fibre Reinforced Polymer Composites.

[26] Nazrin A., Sapuan S.M., Zuhri M.Y.M., Ilyas R.A., Syafiq R., Sherwani S.F.K. (2020). Nanocellulose Reinforced Thermoplastic Starch (TPS), Polylactic Acid (PLA), and Polybutylene Succinate (PBS) for Food Packaging Applications. Front. Chem..

[27] Jumaidin R., Khiruddin M.A.A., Asyul Sutan Saidi Z., Salit M.S., Ilyas R.A. (2020). Effect of cogon grass fibre on the thermal, mechanical and biodegradation properties of thermoplastic cassava starch biocomposite. Int. J. Biol. Macromol..

[28] Sari N.H., Pruncu C.I., Sapuan S.M., Ilyas R.A., Catur A.D., Suteja S., Sutaryono Y.A., Pullen G. (2020). The effect of water immersion and fibre content on properties of corn husk fibres reinforced thermoset polyester composite. Polym. Test..

[29] McDonagh B.H., Chinga-Carrasco G. (2020). Characterization of Porous Structures of Cellulose Nanofibrils Loaded with Salicylic Acid. Polymers (Basel).

[30] Sharma A., Thakur M., Bhattacharya M., Mandal T., Goswami S. (2019). Commercial application of cellulose nano-composites—A review. Biotechnol. Rep..

[31] Abitbol T., Rivkin A., Cao Y., Nevo Y., Abraham E., Ben-Shalom T., Lapidot S., Shoseyov O. (2016). Nanocellulose, a tiny fiber with huge applications. Curr. Opin. Biotechnol..

[32] Syafri E., Sudirman, Mashadi, Yulianti E., Deswita, Asrofi M., Abral H., Sapuan S.M., Ilyas R.A., Fudholi A. (2019). Effect of sonication time on the thermal stability, moisture absorption, and biodegradation of water hyacinth (Eichhornia crassipes) nanocellulose-filled bengkuang (*Pachyrhizus erosus*) starch biocomposites. J. Mater. Res. Technol..

[33] Abral H., Ariksa J., Mahardika M., Handayani D., Aminah I., Sandrawati N., Sapuan S.M., Ilyas R.A. (2019). Highly transparent and antimicrobial PVA based bionanocomposites reinforced by ginger nanofiber. Polym. Test..

[34] Ditzel F.I., Prestes E., Carvalho B.M., Demiate I.M., Pinheiro L.A. (2017). Nanocrystalline cellulose extracted from pine wood and corncob. Carbohydr. Polym..

[35] Rosa M.F.M., Medeiros E.S., Malmonge J.A.J., Gregorski K.S., Wood D.F., Mattoso L.H.C., Glenn G., Orts W.J., Imam S.H. (2010). Cellulose nanowhiskers from coconut husk fibers: Effect of preparation conditions on their thermal and morphological behavior. Carbohydr. Polym..

[36] Ates B., Koytepe S., Ulu A., Gurses C., Thakur V.K. (2020). Chemistry, Structures, and Advanced Applications of Nanocomposites from Biorenewable Resources. Chem. Rev..

[37] Ilyas R.A., Sapuan S.M., Atikah M.S.N., Asyraf M.R.M., Rafiqah S.A., Aisyah H.A., Nurazzi N.M., Norrrahim M.N.F. (2020). Effect of hydrolysis time on the morphological, physical, chemical, and thermal behavior of sugar palm nanocrystalline cellulose (*Arenga pinnata* (Wurmb.) Merr). Text. Res. J..

[38] Ilyas R.A., Sapuan S.M., Ishak M.R., Zainudin E.S. (2019). Sugar palm nanofibrillated cellulose (Arenga pinnata (Wurmb.) Merr): Effect of cycles on their yield, physic-chemical, morphological and thermal behavior. Int. J. Biol. Macromol..

[39] Ilyas R.A., Sapuan S.M., Sanyang M.L., Ishak M.R., Zainudin E.S. (2018). Nanocrystalline cellulose as reinforcement for polymeric matrix nanocomposites and its potential applications: A Review. Curr. Anal. Chem..

[40] Ajao O., Jeaidi J., Benali M., Restrepo A.M., El Mehdi N., Boumghar Y. (2018). Quantification and Variability Analysis of Lignin Optical Properties for Colour-Dependent Industrial Applications. Molecules.

[41] Thielemans W., Can E., Morye S.S., Wool R.P. (2002). Novel applications of lignin in composite materials. J. Appl. Polym. Sci..

[42] Yang H., Yan R., Chen H., Lee D.H., Zheng C. (2007). Characteristics of hemicellulose, cellulose and lignin pyrolysis. Fuel.

[43] Vahabi H., Rastin H., Movahedifar E., Antoun K., Brosse N., Saeb M.R. (2020). Flame Retardancy of Bio-Based Polyurethanes: Opportunities and Challenges. Polymers (Basel).

[44] Rad E.R., Vahabi H., de Anda A.R., Saeb M.R., Thomas S. (2019). Bio-epoxy resins with inherent flame retardancy. Prog. Org. Coat..

[45] Zheng C., Li D., Ek M. (2019). Improving fire retardancy of cellulosic thermal insulating materials by coating with bio-based fire retardants. Nord. Pulp Pap. Res. J..

[46] Sabaruddin F.A., Paridah M.T. (2018). Effect of lignin on the thermal properties of nanocrystalline prepared from kenaf core. IOP Conf. Ser. Mater. Sci. Eng..

[47] Zhang C., Nair S.S., Chen H., Yan N., Farnood R., Li F. (2020). Thermally stable, enhanced water barrier, high strength starch bio-composite reinforced with lignin containing cellulose nanofibrils. Carbohydr. Polym..

[48] Yetiş F., Liu X., Sampson W.W., Gong R.H. (2020). Acetylation of lignin containing microfibrillated cellulose and its reinforcing effect for polylactic acid. Eur. Polym. J..

[49] Ballner D., Herzele S., Keckes J., Edler M., Griesser T., Saake B., Liebner F., Potthast A., Paulik C., Gindl-Altmutter W. (2016). Lignocellulose Nanofiber-Reinforced Polystyrene Produced from Composite Microspheres Obtained in Suspension Polymerization Shows Superior Mechanical Performance. ACS Appl. Mater. Interfaces.

[50] Nair S.S., Kuo P.-Y., Chen H., Yan N. (2017). Investigating the effect of lignin on the mechanical, thermal, and barrier properties of cellulose nanofibril reinforced epoxy composite. Ind. Crops Prod..

[51] Jonoobi M., Harun J., Shakeri A., Misra M., Oksmand K. (2009). Chemical composition, crystallinity, and thermal degradation of bleached and unbleached kenaf bast (*Hibiscus cannabinus*) pulp and nanofibers. BioResources.

[52] Hatakeyama H., Hatakeyama T. (2010). Lignin Structure, Properties and Application. Adv. Polym. Sci..

[53] Zainuddin N., Ahmad I., Kargarzadeh H., Ramli S. (2017). Hydrophobic kenaf nanocrystalline cellulose for the binding of curcumin. Carbohydr. Polym..

[54] Brinchi L., Cotana F., Fortunati E., Kenny J.M. (2013). Production of nanocrystalline cellulose from lignocellulosic biomass: Technology and applications. Carbohydr. Polym..

[55] Jiang W., Han G., Zhou C., Gao S., Zhang Y., Li M., Gong Y., Via B. (2017). The Degradation of Lignin, Cellulose, and Hemicellulose in Kenaf Bast Under Different Pressures Using Steam Explosion Treatment. J. Wood Chem. Technol..

[56] Gan P.G., Sam S.T., Faiq M., Omar M.F. (2019). Thermal properties of nanocellulose-reinforced composites: A review. J. Appl. Polym. Sci..

[57] Rahman R., Hamdan S., Lai J., Hui C. (2017). Differential Scanning Calorimetry (DSC) and Thermogravimetric Analysis (TGA) of Wood polymer nanocomposites. MATEC Web of Conference, Proceedings of the 9th International Unimas Stem Engineering Conference (ENCON 2016) “Innovative Solutions for Engineering and Technology Challenges”, Kuching, Sarawak, 26–28 October 2016.

[58] Chan C.H., Chia C.H., Zakaria S., Ahmad I., Dufresne A. (2013). Production and Characterisation of Cellulose and Nanocrystalline Cellulose from Kenaf Core Wood. BioResources.

[59] Huang S., Zhou L., Li M.C., Wu Q., Zhou D. (2017). Cellulose nanocrystals (CNCs) from corn stalk: Activation energy analysis. Materials (Basel).

[60] Phiri G., Khoathane M.C., Sadiku E.R. (2014). Effect of fibre loading on mechanical and thermal properties of sisal and kenaf fibre-reinforced injection moulded composites. J. Reinf. Plast. Compos..

[61] Guo Y., Zhu S., Chen Y., Li D. (2019). Thermal Properties of Wood-Plastic Composites with Different Compositions. Materials (Basel).

[62] Saba N., Jawaid M., Alothman O.Y., Paridah M.T. (2016). A review on dynamic mechanical properties of natural fibre reinforced polymer composites. Constr. Build. Mater..

[63] Rozman H.D., Tan K.W., Kumar R.N., Abubakar A., Ishak Z.A.M., Ismail H. (2000). The effect of lignin as a compatibilizer on the physical properties of coconut fiber–polypropylene composites. Eur. Polym. J..

[64] Mirjalili F., Chuah L., Salahi E. (2014). Mechanical and Morphological Properties of Polypropylene/Nano *α*-Al_2_O_3_ Composites. Sci. World J..

[65] Guo C., Song Y., Wang Q., Shen C.-S. (2006). Dynamic-mechanical analysis and SEM morphology of wood flour/polypropylene composites. J. For. Res..

[66] Yousefian H., Rodrigue D. (2016). Effect of nanocrystalline cellulose on morphological, thermal, and mechanical properties of Nylon 6 composites. Polym. Compos..

[67] Mofokeng J.P., Luyt A.S., Kovacs J.G. (2014). Comparison of injection moulded, natural fibre reinforced composites with Comparison of injection moulded, natural fibre-reinforced composites with PP and PLA as matrices. J. Thermoplast. Compos. Mater..

[68] Subasinghe A.D.L., Das R., Bhattacharyya D. (2015). Fiber dispersion during compounding/injection molding of PP/kenaf composites: Flammability and mechanical properties. Mater. Des..

[69] Chapple S., Anandjiwala R. (2010). Flammability of natural fiber-reinforced composites and strategies for fire retardancy: A review. J. Thermoplast. Compos. Mater..

